# Affective and Enjoyment Responses to Sprint Interval Training in Healthy Individuals: A Systematic Review and Meta-Analysis

**DOI:** 10.3389/fpsyg.2022.820228

**Published:** 2022-03-09

**Authors:** Mingzhu Hu, Mary E. Jung, Jinlei Nie, Zhaowei Kong

**Affiliations:** ^1^Faculty of Education, University of Macau, Macao, Macao SAR, China; ^2^School of Health and Exercise Sciences, University of British Columbia, Okanagan, BC, Canada; ^3^School of Health Sciences and Sports, Macao Polytechnic Institute, Macao, Macao SAR, China

**Keywords:** all-out, high-intensity interval training (HIIT), psychological responses, affect (emotion), enjoyment, perception, exercise adherence

## Abstract

Sprint interval training (SIT) is characterized by intensity of “all-out” effort and superior time-efficiency compared to traditional moderate-intensity continuous training (MICT) and has been proposed as one viable solution to address the commonly reported barrier of lack of time for physical activity. While substantial physiological benefits of participation in SIT have been well-documented, the psychological responses to SIT are less clear. No systematic review has been conducted thus far to respond to the assumption that its supramaximal intensity will induce adverse feelings. Therefore, the aim of the present study was to synthesize studies analyzing affective and enjoyment responses to SIT and to compare the responses to SIT with MICT and other high intensity interval training (HIIT) protocols with lower intensities. After searching relevant databases up until 22nd March 2021, twenty-five studies meeting the inclusion criteria were included in the present review. Random effect meta-analysis using the pooled data demonstrated that SIT induced similar post-exercise affective valences during the training compared to MICT and HIIT, but lower affective valences immediately post-exercise compared to MICT. Moreover, affective responses during SIT decreased to negative valences according to the results from most included studies, while low-volume SIT protocols with shorter sprint duration and repetitions induced more positive affective responses. Level of enjoyment after SIT were positive and were comparable to MICT or HIIT. Overall, the results from the existing literature indicate that SIT might cause unpleasant feelings during the training and be perceived less pleasurable than MICT immediately post training but could be a comparably enjoyable modality for healthy individuals in relation to MICT or HIIT, despite its supramaximal intensity. Low-volume SIT may be a realistic option for individuals seeking a time-efficient workout with comparable affective responses to MICT or HIIT.

**Systematic Review Registration:**

[https://www.crd.york.ac.uk/PROSPERO], Identifier [CRD42021284898].

## Introduction

Regular participation in physical activity has been recommended as an essential means to achieve and maintain good health status. Sedentary behavior is linked to several deleterious health consequences including increased metabolic risk, vascular defects and decreased bone mineral content (Tremblay et al., 2010). However, there are still a tremendous number of individuals worldwide who remain inactive and fail to meet physical activity guidelines written by WHO or national departments (Sallis et al., 2016; Guthold et al., 2018). Current literature suggests that perceived lack of time remains the most frequently reported barrier to participating in physical activity regularly (Trost et al., 2002; Pagnan et al., 2017). High-intensity interval training (HIIT) has been promoted consistently in the literature, in part because of its time-efficiency, but mainly due to its comparable effects on improving health and fitness compared with traditional cardiovascular training (i.e., moderate intensity continuous exercise, MICT) (Biddle and Batterham, 2015; Milanović et al., 2015; Batacan et al., 2017). HIIT is characterized by repeated short high-intensity bouts separated with either active or inactive recovery periods. More recently, sprint interval training (SIT) has generated great interest and popularity, as SIT further enhances the time-efficiency of HIIT by shortening the interval duration with higher intensity “all-out” sprints (i.e., ≤30 s in duration) compared with HIIT while maintaining the beneficial physiological adaptations (Buchheit and Laursen, 2013). Several systematic reviews and meta-analyses have demonstrated the potential advantages of participating in SIT for improving physical health such as cardiorespiratory fitness (Gist et al., 2014; Vollaard et al., 2017; Wen et al., 2019), body composition (Keating et al., 2017) and metabolic adaptions (Kessler et al., 2012; Jelleyman et al., 2015) with much less time investment compared with MICT and HIIT.

Although previous studies have reported similar or greater improvements in health status after engaging in SIT compared with more traditional training methods such as MICT (Kessler et al., 2012; Gist et al., 2014; Weston et al., 2014; Jelleyman et al., 2015; Keating et al., 2017; Way et al., 2019), many have been skeptical on the practicality of both HIIT and SIT for the general population. Critics argue that the complexity and strenuous nature of HIIT or SIT could require a high level of motivation, self-regulation, and self-efficacy (Hardcastle et al., 2014). Moreover, supramaximal intensity in SIT has been feared to potentially lead to adverse affective responses, consequently discouraging future exercise adherence (Haile et al., 2016), especially when SIT is performed by inactive individuals with low fitness levels and limited exercise experiences. In response to critiques to HIIT, several meta-analyses and systematic reviews have been conducted to generalize findings of psychological responses to HIIT and answer the doubts of implementing HIIT protocols (Stork et al., 2017; Oliveira et al., 2018; Niven et al., 2020). The results in the reviews indicated that acute enjoyment responses to HIIT is similar or greater compared to MICT protocols, yet there could be more aversive affective valences to HIIT compared to MICT. The differences between measuring focuses of enjoyment (i.e., affective judgments involving cognitive processing) and core affect (i.e., immediate feelings of pleasure or unpleasure) (Stevens et al., 2020) might explain the discrepant findings regarding enjoyment and affect valences in HIIT. However, to the best of our knowledge, none of previous analyses have focused exclusively on SIT protocols and explored both long-term and acute responses to SIT. Considerations of distinguishing SIT with all-out efforts from other HIIT protocols with near maximal efforts could be important, as SIT could induce larger neuromuscular load and dependence on anaerobic metabolism (Buchheit and Laursen, 2013), which may lead to different physiological and psychological responses in SIT compared to lower intensity HIIT (Hall et al., 2002). In fact, the debate of public implications of HIIT seems to be more intense when referring to SIT with “all-out” efforts above the ventilatory threshold (Hardcastle et al., 2014) which theoretically could trigger negative feelings during exercise according to the dual-mode theory (Ekkekakis, 2003). Moreover, effects of long-term SIT interventions on affective responses would be worth of analyzing considering that meaningful physiological improvements (e.g., cardiorespiratory fitness) take time to generate.

Given that the beneficial effects of SIT protocols on health parameters, but the psychological responses to SIT are still under debate, the present systematic review aimed to locate all related SIT studies in the literature and to comprehensively evaluate the affective and enjoyment responses to SIT in healthy populations with respect to MICT and other HIIT protocols. Affective responses (AR) (e.g., enjoyment, affect) were primary constructs under investigation, as AR have been frequently analyzed and suggested to be associated with future exercise behaviors in the current literature (Rhodes and Kates, 2015; Brand and Ekkekakis, 2018). Cognitive parameters which might affect AR, such as self-efficacy, self-reported tolerance and preference of exercise were secondary variables of interest. Given considerable differences and inconsistency in SIT protocol designs (i.e., varying interval and recovery durations, ratios of interval to recovery lengths, number of the sprint bouts and total exercise time) in the included studies, a high heterogeneity in the meta-analysis was anticipated as reported in previous meta-analyses (i.e., the between-study variance (I^2^) ranged from 75 to 95%) (Oliveira et al., 2018; Niven et al., 2020), which could limit the validity of the analysis. Therefore, the present study aimed to distinguish the variations between SIT protocol designs and narratively synthesize the correspondent AR, psychological, and behavioral responses.

## Methods

The current study was undertaken in accordance with the Preferred Reporting Items for Systematic Reviews and Meta-Analyses (PRISMA) guidelines and was registered with the PROSPERO database (CRD42021284898). For the purpose of identifying all studies that have examined AR responses (e.g., affect, enjoyment) to SIT protocols, the following electronic databases were searched: Web of Science, PubMed, Scopus, ScienceDirect, NCBI, Cochrane, Medline. Search strategy were adapted for each database using combinations of independent and dependent variable keywords to identify relevant available studies published until 22th March 2021. Combinations of the following keywords were used with “all fields”: “sprint interval training” OR “all-out” OR “sprint,” OR “SIT” AND “perception” OR “enjoyment” OR “enjoy*” OR “pleasure” OR “affect” OR “affective responses” OR “exercise induced feeling state” OR “subjective exercise experience” OR “pleasant feeling state.” Reference lists in recent reviews and meta-analyses were also searched to find relevant studies.

### Eligibility Criteria

The following inclusion criteria was applied for selection of studies: (1) published in English; (2) analyzed healthy human participants older than 18 years old who could be of both sexes, physically active or sedentary, with normal weight or excess weight including overweight and obese subjects with no pre-existing health conditions; (3) included SIT protocols are characterized with work intervals with supramaximal intensity or “all-out” efforts in either short (≤10 s) or long (10–30 s) intervals and performed either in the laboratory or unsupervised condition in any training modality. (4) With comparison groups of MICT and/or HIIT and/or SIT protocols with different designs or same SIT protocols applied in varied subjects (e.g., subjects with low fitness level vs. high fitness level). (5) Reported acute or long-term psychological responses with primary outcomes of affect measured by the Feeling Scale (FS) (Hardy and Rejeski, 1989) and/or enjoyment measured by the Physical Activity Enjoyment Scale (PACES) (Kendzierski and DeCarlo, 1991) or the Exercise Enjoyment Scale (EES) (Stanley and Cumming, 2010) with or without other psychological outcomes such as arousal measured by the Felt of Arousal Scale (FAS) (Svebak and Murgatroyd, 1985), preference and self-efficacy and intentions to SIT protocols; (6) randomized or non-randomized controlled experimental trials using between-subject or within-subject design and having gone through scientific peer-review. Studies that applied SIT protocols in conjunction with other interventions (e.g., dietary interventions) were excluded for the purpose of the current study. Importantly, HIIT protocols in the current review were classified as interval training protocols with near maximal intensity intervals (e.g., elicits 90% VO_2max_) lasting longer than 30 s (Keating et al., 2017; MacInnis and Gibala, 2017), while MICT protocols were defined as continuous exercise at a moderate intensity (46- 64% VO_2max_/HR_*max*_ or 40%–60% HR reserve/VO_2_ reserve according to ASCM guidelines (Pescatello et al., 2014).

### Studies Selection and Data Extraction

**FIGURE 1:**
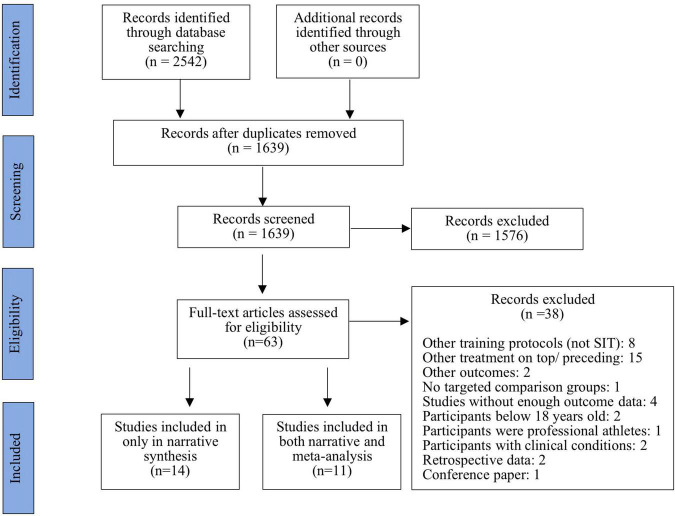
Flow diagram of screening and selection of articles for review.

All eligible studies were recorded using an electronic data extraction form. Studies after duplicates removed were screened by titles, followed by abstract screening in order to further exclude studies that did not meet the inclusion criteria. Full texts were screened to make the final decision of inclusion when studies were eligible after title and abstract screening (Figure 1). The extracted data from the final eligible studies included characteristics of participants, study design, study measurements, study results (i.e., means and standard deviations for each experimental group), and analyses. Authors of the included studies were contacted if no direct data available.

### Risk of Bias

**FIGURE 2:**
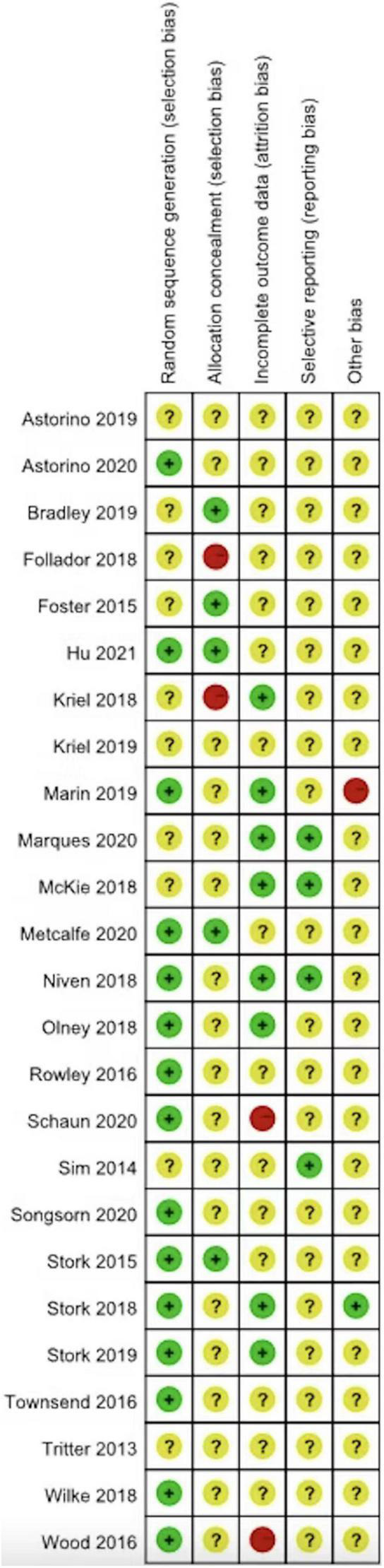
Individual study of risk of bias assessment.

Cochrane Risk of Bias tool (Higgins et al., 2011) was used to assess risk of bias in included studies (Figure 2). Risk of bias for blinding of participants and personnel or blinding of outcome assessment were not included for the assessment as the studies in the current review could not possibly meet these criteria due to the nature of study protocols. A visual analysis of the created funnel plot was performed to identify publication bias based on evidence of asymmetry. Heterogeneity was calculated using I^2^ tests with interpretation of high (I^2^ = 75%), moderate (I^2^ = 50%) and low (I^2^ < 25%) heterogeneity (Higgins et al., 2019).

### Analysis

Affective responses were analyzed through the Feeling Scale (FS) and the Felt of Arousal Scale (FAS), while enjoyment responses were analyzed through the Physical Activity Enjoyment Scale (PACES) and the Exercise Enjoyment Scale (EES). Both the FS and the FAS are single item scale with a 11-point item ranging from −5 (very bad) to + 5 (very good) or a 6-point item scale demonstrating low activation (1 point) to high activation (6 points), respectively. The FS scores demonstrated core affective responses to exercise using simple judgment of good or bad. When combining with the FAS scores with the FS scores, specific affective state could be captured through circumplex model of affect. Specifically, high arousal and positive affect indicates a sense of energy, while low arousal and positive affect indicates a sense of calmness. Negative affect with either low arousal or high arousal indicates depression or distress, respectively. The EES is a single item, 7-point scale indicating an overall feeling of enjoying or not enjoying, which mostly used during acute exercise bout due to its simplicity. The PACES questionnaire is a 7-point 18-item bipolar scale (11 items are scored reversely) with scores ranging from 18 to 126, in which respondents recall the feelings and enjoyment level toward the exercise they have done.

**FIGURE 3:**
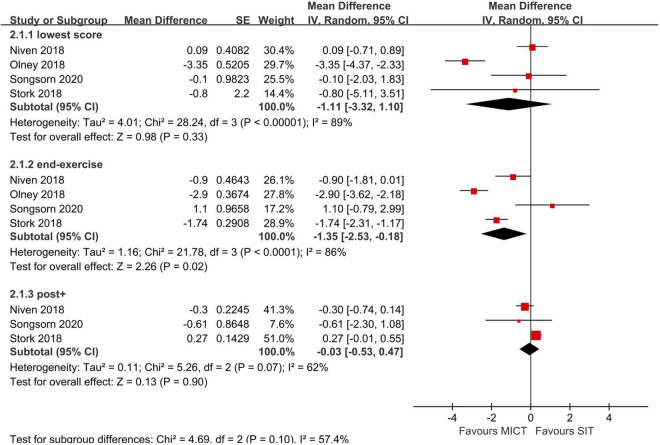
Random effects meta-analyses for the influence of sprint interval training (SIT) vs. moderate-intensity continuous training (MICT) on affect.

**FIGURE 4:**
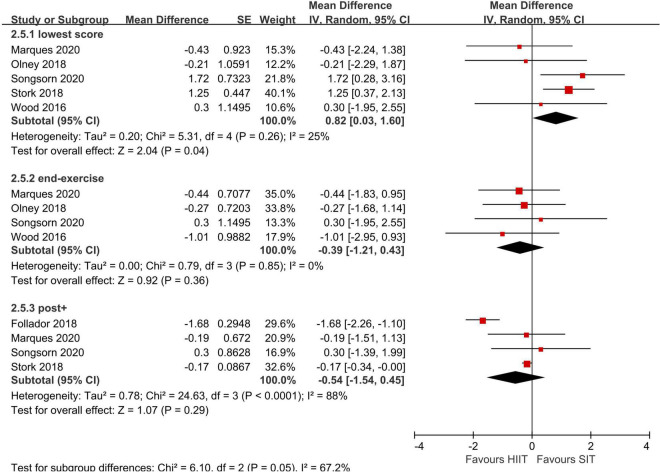
Random effects meta-analyses for the influence of sprint interval training (SIT) vs. high-intensity interval training (HIIT) on affect.

Meta-analyses were conducted using the Review Manager 5 (version RevMan 5.4). We compared post enjoyment responses with data extracted from the PACES and in-task and post affect valences with data extracted from the FS between MICT and SIT, HIIT and SIT. Comparisons were made in the lowest scores of affect valences, affect valences immediately postexercise (labeled as “end-exercise” in Figures 3, 4), as these two variables has been suggested as the most meaningful parameters of affective responses (Stork et al., 2018). Moreover, FS scores post exercise > 5 min (labeled as “post+” in the figures) following completion of exercises were also compared considering the rebound effects. Given the methodology differences in the included studies, the generic inverse-variance method with a random effects model was implemented to estimate the effect sizes (ESs) and their 95% confidence intervals (95% CI) through mean difference and standard error. Sensitivity analyses were performed to examine if the results were largely impacted by a single study with large weight.

Included studies were categorized into four groups based on the protocol designs: (1) classic SIT with 4–6 repetitions of 30-s sprints with 4-min active or passive recovery periods (Burgomaster et al., 2006; Gibala et al., 2006); (2) REHIT (reduced-exertion high intensity interval training) with 2–3 repetitions of 20 s sprints interspersed with 2–4 min active or passive recovery periods (Astorino et al., 2020); (3) RST (repeated-sprint training) with ≥ 10 repetitions of ≤ 10-s sprints interspersed with ≤ 60-s active or passive recovery (Marques et al., 2020); (4) Tabata SIT with 7–8 repetitions of 20-s sprints interspersed with 10-s active or passive recovery periods (Tabata, 2019). It should be mentioned that the terminologies used in the categorization was not strictly consistent with the original SIT designs. For example, some studies that made small modifications (i.e., 8 repetitions of 30-s sprints instead of the original 4 repetitions or 6 repetitions of 20-s sprints instead of the original 30-s) to the original prescriptions of classic SIT would still be categorized into classic SIT group but termed as “modified classic SIT” based on similarities on total interval duration, rest duration, and the number of interval repetitions. Moreover, RST which often used as an effective training method in athletes is characterized with sprints lasting from 3 to 7 s according to previous studies (Buchheit and Laursen, 2013; Marques et al., 2020), yet the categorization in the present study included SIT protocol with ≤ 10-s sprints. Range of all reported scores, peak negative responses, change scores and corresponded SIT protocol design would be one of the interests of the narrative analysis. Moreover, changes in long-term psychological responses to SIT would be narratively analyzed if data available.

## Results

A total of 2,542 studies were identified from the database after initial search. After de-duplication and title and abstract screening, 60 studies were retrieved and screened by full-text, and 25 studies were included in the current review (Tritter et al., 2013; Sim et al., 2014; Foster et al., 2015; Stork et al., 2015, 2018, 2019; Kong et al., 2016; Wood et al., 2016; Rowley et al., 2017; Townsend et al., 2017; Follador et al., 2018; Kriel et al., 2018, 2019; McKie et al., 2018; Niven et al., 2018; Olney et al., 2018; Astorino et al., 2019, 2020; Bradley et al., 2019; Marin et al., 2019; Wilke et al., 2019; Marques et al., 2020; Metcalfe et al., 2020; Schaun and Alberton, 2020; Songsorn et al., 2020; Hu et al., 2021). A flow chart presented the Figure 1 demonstrated the completed steps of selections of the included studies.

### Study Characteristics

Details of the characteristics of the included studies are summarized in Table 1. A total of 675 healthy adult individuals (337 men and 338 women) participated in the included experiments. Most of the studies (i.e., 20 out of 25 studies) recruited young participants below 30 years old. Close to half of the studies (i.e., 11 out of 25 studies) involved active participants with high cardiorespiratory fitness level indicated by above average VO_2max_ ranging from 40–55 ml kg^–1^ min^–1^. Three studies analyzed affective responses from overweight females (Rowley et al., 2017; Astorino et al., 2019) and males (Sim et al., 2014) and one study from women with excess weight (Hu et al., 2021), whereas all the other studies focused on normal weight participants.

**TABLE 1:** Participants’ characteristics of all included studies.

Study	N/Gender/Grouping	Age	BMI (kg/m^2^)	VO_2max_ (ml kg^–1^ min^–1^)	Physical activity level
Astorino et al., 2019	Total: 19 F	37.5 ± 10.5	39.0 ± 4.3		Inactive and obese
	Periodized interval training: *n* = 9	37 ± 12		17 ± 2	
	HIIT: *n* = 10	37 ± 8		*21 ± 3	
Astorino et al., 2020	Total: 85 (*M* = 44, *F* = 41)				Active and inactive
	SIT (subjects below average VO_2max_): *n* = 43 (*M* = 19, *F* = 24)	23.6 ± 6.5	23.0 ± 2.2	33 ± 5	
	SIT (subjects above average VO_2max_): *n* = 42 (*M* = 25, *F* = 17)	22.8 ± 3.6	26.4 ± 3.9	*41 ± 6	
Bradley et al., 2019	Total: 36 (*M* = 12, *F* = 24)	21 ± 2			Active and inactive
	SIT (LT-low tolerance): *n* = 17 (*M* = 5, *F* = 12);		∼23.9	49.3 ± 4.2	
	(further subdivided into very low tolerance group, *n* = 9)				
	SIT (HT-high tolerance): *n* = 19 (*M* = 7, *F* = 12)		∼23.6	54.8 ± 1.8	
Follador et al., 2018	Total: 14 M	23.4 ± 2.8	24.5 ± 2.9	45.8 ± 4.8 (cycling)	Active
				49.9 ± 5.6 (running)	
Foster et al., 2015	Total: 55 (*M* = 17, *F* = 38)				Inactive
	SIT (Tabata): *n* = 21	20.3 ± 2.1 (M); 19.5 ± 1.2 (F)	∼26.5 (M); ∼23.9 (F)	34 ± 6.5	
	MICT (Steady- state): *n* = 19	19.5 ± 1.4 (M); 19.6 ± 2.9 (F)	∼28.5 (M); ∼25.4 (F)	33.6 ± 5.4	
	HIIT (Meyer): *n* = 15	19.3 ± 1.3 (M); 19.9 ± 2.8 (F)	∼28.5 (M); ∼25.4 (F)	34.3 ± 9.1	
Hu et al., 2021	Total: 60 F	21.2 ± 1.4	26.0 ± 3.0		Inactive
	SIT: *n* = 15		25.6 ± 2.3	30.8 ± 3.7	
	MICT: *n* = 15		25.8 ± 2.6	30.6 ± 3.5	
	HIIT: *n* = 15		25.5 ± 2.4	31.6 ± 2.2	
	CON: *n* = 15		25.9 ± 2.4	28.8 ± 3.6	
Kriel et al., 2018	Total: 12 M	24 ± 3.0	∼25.5	43.5 ± 4.3	Inactive
Kriel et al., 2019	Total: 11 M	23 ± 4.0	∼24.4	40.7 ± 4.3	Inactive
Marin et al., 2019	Total: 14 M	24 ± 4.0	26.2 ± 2.7		Active
Marques et al., 2020	Total: 23 (*M* = 11, *F* = 12)	25.6 ± 4.8(*M*)	∼23.1 (M)		Inactive
		25.0 ± 3.5(*F*)	∼22.6 (F)		
McKie et al., 2018	Total: 43 (*M* = 27, *F* = 16)				Active
	SIT (30 s): *n* = 11 (*M* = 6, *F* = 5)	21.0 ± 1.7	∼24.05	46.3 ± 8.4	
	SIT (15 s): *n* = 11 (*M* = 7, *F* = 4)	20.4 ± 1.9	∼25.03	46.8 ± 7.1	
	SIT (5 s): *n* = 12 (*M* = 7, *F* = 5)	19.4 ± 1.1	∼24.29	46.2 ± 7.3	
	CON: *n* = 9 (*M* = 7, *F* = 2)	23.1 ± 2.0	∼24.18	50.3 ± 5.7	
Metcalfe et al., 2020	Total: 25 (*M* = 12, *F* = 13)	47 ± 9	27.5 ± 4.4	28 ± 7	Inactive
	HIIT: *n* = 13 (*M* = 6, *F* = 7)				
	CON: *n* = 12 (*M* = 6, *F* = 6)				
Niven et al., 2018	Total:12 M	25 ± 7	∼24.4	48.2 ± 6.7	Active
Olney et al., 2018	Total: 19 (*M* = 10, *F* = 9)	24.0 ± 3.3	23.1 ± 3.9	42.6 ± 6.5(*M*)	Active
				38.0 ± 4.2(*F*)	
Rowley et al., 2017	Total: 12 F	34.1 ± 6.1	31.3 ± 6.8	27.0 ± 6.2	Inactive and overweight
	SIT: *n* = 5	37 ± 6	32.3 ± 4.7	29.5 ± 3.3	
	MICT: *n* = 7	30 ± 4	29.5 ± 1.8	26.7 ± 2.4	
Schaun and Alberton, 2020	Total: 41 M	23.7 ± 0.7	∼24.5	46.7 ± 7.3	Active
	SIT (T- treadmill): *n* = 15				
	SIT (WB- whole body): *n* = 12				
	MICT: *n* = 14				
Sim et al., 2014	Total: 17 M	30.0 ± 8.0	27.7 ± 1.6	39.2 ± 4.8	Inactive and overweight
Songsorn et al., 2020	Total: 8#	21 ± 1	24.9 ± 2.1	39 ± 10	Inactive and active
Stork et al., 2015	Total: 20 (*M* = 10, *F* = 10)	22 ± 4			Active
Stork et al., 2018	Total: 30 (*M* = 12, *F* = 18)	21.23 ± 3.81	22.47 ± 3.02	31.3 ± 6.2	Inactive
Stork et al., 2019	Total: 24 (*M* = 12, *F* = 12)	24.08 ± 4.61	23.09 ± 2.68	39.2 ± 8.5	Inactive
Townsend et al., 2017	Total: 9 M	23.3 ± 3.0	22.4 ± 2.2	48.9 ± 5.3	Active
Tritter et al., 2013	Total: 74 (*M* = 32, *F* = 42)				Active
	SIT (high efficacy): *n* = 25	20.9 ± 1.7	23.7 ± 3.3		
	SIT (low efficacy): *n* = 25	21.8 ± 2.4	25.8 ± 3.5		
	SIT (control): *n* = 24	22.6 ± 2.3	24.4 ± 3.2		
Wilke et al., 2019	Total: 33 (*M* = 12, *F* = 21)	25 ± 5	24 ± 4		Inactive
	SIT (Tabata):20	26 ± 6	23 ± 4		
	MICT: 13	24 ± 3	24 ± 4		
Wood et al., 2016	Total: 12 (*M* = 8, *F* = 4)	24.2 ± 6.2	∼23.9	40.6 ± 4.3	Active

*, significantly different between experimental groups; ∼, data not available initially and was calculated using mean value reported in the studies; #, only data from healthy participants were extracted; BM, body mass; F, female; M, male; N, the number of participants.

Regarding the study design (see Supplementary Table 1), half of the studies (*n* = 13) used a randomized cross-over study design, while the remaining studies incorporated a randomized between-participants study design. More than half of the included studies (*n* = 17) reported acute responses to SIT. Other eight studies implemented intervention lasting 4–12 week (4-week, *n* = 1; 6-week, *n* = 3; 8-week, *n* = 1; 12-week, *n* = 2; 16-week, *n* = 1). Most studies (*n* = 23) conducted experiments in the laboratory. Three studies involved outdoor exercise under supervision (Wilke et al., 2019; Marques et al., 2020; Schaun and Alberton, 2020). Only two out of 25 studies analyzed unsupervised SIT intervention (Astorino et al., 2019; Metcalfe et al., 2020) and one study reported 4-week follow-up exercise behavior in unsupervised condition (Stork et al., 2018).

Regarding the SIT protocols selected by the included studies, more than half of the studies (*n* = 13) included a classic Wingate SIT protocol with four sets of 30-s all-out sprints. Three studies analyzed REHIT with shortened 20-s all-out sprints compared to classic SIT. Four studies incorporated Tabata SIT, while seven studies analyzed RST. Total sprint duration varied in the included SIT protocols, which ranged from 20-s to 10-min. Total training durations (including recovery intervals) in most of the SIT protocols adopted in the included study (i.e., 22 out of 25) were less than 20-min, which were significantly shorter than most of the studies compared MICT (i.e., 30–65 min) (9 out 11 studies) or HIIT protocols (i.e., 20–60 min) (8 out 10 studies). Most studies used cycling (*n* = 18) or running (*n* = 8) (Hardy and Rejeski, 1989; Foster et al., 2015; Rhodes and Kates, 2015; Bradley et al., 2019; Tabata, 2019) as the training mode. Three studies adopted other less frequently used training mode, which involved battling rope exercise (Marin et al., 2019) and whole-body calisthenics (Wilke et al., 2019; Schaun and Alberton, 2020).

### Affective Responses

A total of 11 studies (Stork et al., 2015, 2018; Wood et al., 2016; Townsend et al., 2017; Follador et al., 2018; Niven et al., 2018; Olney et al., 2018; Bradley et al., 2019; Marin et al., 2019; Astorino et al., 2020; Marques et al., 2020) recorded acute affect responses to SIT using the Feeling Scale (FS) (Hardy and Rejeski, 1989). Involved SIT protocols were classic SIT (*n* = 6) (Stork et al., 2015; Wood et al., 2016; Townsend et al., 2017; Olney et al., 2018; Marin et al., 2019; Marques et al., 2020), REHIT (*n* = 2) (Stork et al., 2018; Astorino et al., 2020), RST (*n* = 5) (Townsend et al., 2017; Niven et al., 2018; Bradley et al., 2019; Marin et al., 2019; Marques et al., 2020), and Tabata (*n* = 1) (Follador et al., 2018). The lowest reported mean affective valences ranged from −2.8 to 1.5. Most of the studies (i.e., 8 out of 12 studies) reported the lowest affective response in the SIT condition. The lowest mean affect was observed in classic SIT protocols (i.e., −2.8 ± 2.5) (i.e., 4 s × 30 s sprinting + 240 s recovery) (Townsend et al., 2017) (2.0 ± 2.5) (i.e., 8 s × 30 s sprinting at intensity of 130% Wmax + 90 s active recovery at 25% Wmax) (Wood et al., 2016). The four studies that reported positive lowest affect incorporated REHIT (i.e., 2 s × 20 s sprinting against 5% of BM + 3-min recovery) (Astorino et al., 2020) or RST (i.e., 10 s × 6 s sprinting against 7.5% of BM + 60 s passive recovery) (Niven et al., 2018) or classical SIT with background music playing (Stork et al., 2015) (i.e., 4 s × 30 s sprinting against 7.5% of their body weight + 4-min recovery). All studies that recorded in-task affective valences reported a trend of declining FS scores during SIT (Stork et al., 2015, 2018; Wood et al., 2016; Townsend et al., 2017; Niven et al., 2018; Olney et al., 2018; Astorino et al., 2019, 2020; Bradley et al., 2019; Marin et al., 2019; Marques et al., 2020). As would be predicted, there were rebounds in the FS scores in studies that also recorded post-training (>1 min) affect (Stork et al., 2015, 2018; Townsend et al., 2017; Niven et al., 2018; Marin et al., 2019; Marques et al., 2020).

Four studies compared SIT to MICT in acute affective responses (Niven et al., 2018; Olney et al., 2018; Stork et al., 2018; Songsorn et al., 2020) in scores of the FS and the results were pooled to conduct meta-analyses. Meta-analyses showed no significant effect in favor of either MICT or SIT in the lowest score during exercise and post exercise (Figure 3). However, the meta-analysis demonstrated a large effect in favor of MICT in FS scores recorded immediately post-exercise. A high degree of heterogeneity existed in the meta-analysis of the lowest score (I^2^ = 89%) and end-exercise score (i.e., immediately post exercise) (I^2^ = 86%) and a moderate to high degree of heterogeneity in the analysis of the post-exercise score (I^2^ = 62%).

Significantly more negative scores (*p* ≤ 0.05) during SIT compared to MICT were reported in one study that incorporated classic SIT (i.e., 6 s × 20 s at intensity of 140% W_*max*_ + 75 s active recovery at intensity of 20% W_*max*_ (Olney et al., 2018), while the other two studies that analyzed RST (i.e., 10 s × 6 s sprinting against 7.5% of BM + 60 s passive recovery) (Niven et al., 2018) and REHIT (i.e., 3 s × 20 s sprinting against 5% of BM + 120 s passive recovery) (Stork et al., 2018) showed no significant differences in affect between SIT and MICT during the exercise. However, larger reduction of affect valences in SIT than MICT from baseline to immediately end training were observed in all the three studies.

Six studies compared SIT to HIIT in acute affect valences (Wood et al., 2016; Follador et al., 2018; Olney et al., 2018; Stork et al., 2018; Marques et al., 2020; Songsorn et al., 2020). No significant differences in affect valences recorded immediately post-training or > 5-min post training between SIT and HIIT were found in the meta-analyses. Nevertheless, in the study of Follador (Follador et al., 2018), affect valences measured 10-min post training was higher in HIIT (2.1 ± 2.0) compared to Tabata SIT (−1.1 ± 2.5) and classic SIT (0.4 ± 2.9). There was a large effect in favor of HIIT compared to SIT in the lowest score during exercise, suggesting a larger adverse effect of SIT on affect state. In the analysis of end-exercise, extracted data from the study of Stork et al. (2018) was excluded due to potentially driven effect of meta-analysis from the large weight (weight = 78.5%). Sensitivity analysis showed that there were still no significant differences in affect valences immediately post training between SIT and HIIT before removal of the study by Stork et al. (2018) (*Z* = 0.07, *p* = 0.95, CI: −0.39 to 0.37). The degree of heterogeneity was high in the post exercise FS score (I^2^ = 88%) but low in the lowest score (I^2^ = 25%) and end-exercise (i.e., immediately post exercise) score (I^2^ = 0%).

Combing affect and perceived activations (i.e., the circumplex model) could provide a broader and more accurate understanding of affect state (Ekkekakis, 2008), yet only five out of 25 included studies recorded perceived activations through the felt of arousal scale (FAS), and two out of the four studies reported and discussed circumplex model in their articles as presented in Supplementary Table 1 (Niven et al., 2018; Bradley et al., 2019). Three studies analyzed RST (i.e., 10 s × 6 s sprinting against 7.5% BM + 60 s passive recovery (Niven et al., 2018) or active recovery (Bradley et al., 2019) or 12 s × 10 s sprinting + 30 s recovery (Marin et al., 2019), and two analyzed REHIT (i.e., 3 s × 20 s sprinting against 5% of BM + 120 s passive recovery (Stork et al., 2018, 2019). Perceived activation increased significantly during SIT with mean peak valences ranged from 3.0 to 4.5 in SIT. One study compared SIT with MICT in perceived activations, with results showing a significant higher perceived activation in SIT than MICT.

### Enjoyment Responses

A total of 17 studies (Tritter et al., 2013; Sim et al., 2014; Stork et al., 2015, 2018, 2019; Rowley et al., 2017; Townsend et al., 2017; Kriel et al., 2018, 2019; McKie et al., 2018; Olney et al., 2018; Astorino et al., 2019, 2020; Marin et al., 2019; Marques et al., 2020; Schaun and Alberton, 2020; Hu et al., 2021) reported post-exercise enjoyment responses to SIT based on the PACES. Four out of the 17 studies (Townsend et al., 2017; McKie et al., 2018; Marin et al., 2019; Metcalfe et al., 2020) used a modified version of the PACES with one 7-point item removed (i.e., current absorption in the activity) and the highest score of 119 instead of the original highest score of 126. Moreover, two of the three studies using modified PACES added two separated items (i.e., “how much did you enjoy?” and “enjoyment performing 3 × /week for next month”). One out of the 18 studies used a PACES of sixteen 5-point items (a total score of 80) (Rowley et al., 2017). The average percentage of PACES score ranged from 51 to 81%. Both the lowest (53%) (McKie et al., 2018) and the highest enjoyment level (81%) were reported in RST protocols (Townsend et al., 2017). There were large inconsistencies in the measurement time of the PACES across studies (i.e., immediately post: *n* = 6; within 5-min end of training: *n* = 2; 5-min post training: *n* = 3; 10-min post training: *n* = 3; 15-min post training: *n* = 1; 30-min post training: *n* = 2; 60-min post training: *n* = 1). In the study of Stork et al. (2018), PACES scores were recorded immediately post-training, 30-min post training and 60-min post training, and the main effects of time showed significant increased enjoyment indicating a rebound effect.

**FIGURE 5:**
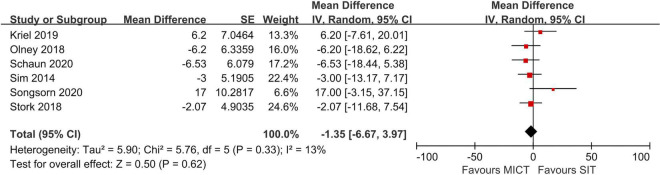
Random effects meta-analyses for the influence of sprint interval training (SIT) vs. moderate-intensity continuous training (MICT) on enjoyment.

Seven studies compared SIT with MICT in acute enjoyment responses based on the PACES (Sim et al., 2014; Olney et al., 2018; Stork et al., 2018; Kriel et al., 2019; Marques et al., 2020; Schaun and Alberton, 2020; Songsorn et al., 2020). The results of meta-analysis showed that there was no significant effect in favor of either SIT or MICT (*Z* = 0.50, *P* > 0.05, CI: −6.67 to 3.97) (Figure 5). Most of the studies (*n* = 6) reported similar post-exercise enjoyment in SIT regardless the protocol design and measurement time (i.e., included classic SIT, REHIT, RST, Tabata) compared with MICT. However, one study (Kriel et al., 2019) that analyzed work-matched SIT and MICT reported significantly lower enjoyment level in a classic SIT session (Kriel et al., 2018). Moreover, in the study of Foster (Foster et al., 2015) which recorded enjoyment using the EES, pre-, during- and post- training, significant lower enjoyment in a Tabata session (i.e., 8 sprints of 20 s at 170% VO_2max_ interspersed with 10 s rest) compared to MICT was reported.

**FIGURE 6:**
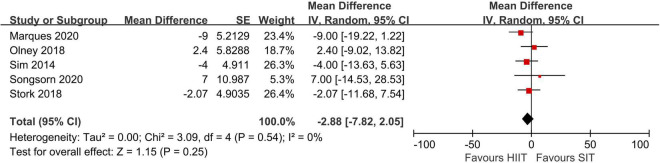
Random effects meta-analyses for the influence of sprint interval training vs. high-intensity interval training on enjoyment. Effect sizes are shown as mean difference and 95% confidence intervals.

Five studies (Sim et al., 2014; Olney et al., 2018; Stork et al., 2018; Marques et al., 2020; Songsorn et al., 2020) compared enjoyment responses to SIT protocols (included classic SIT, REHIT, and RST) with HIIT protocols using PACES, while one study which analyzed Tabata used the EES (Foster et al., 2015). For studies that incorporated more than one HIIT protocols, results from the most identical protocol to protocols in other studies (i.e., 60 s interval durations) were used in the meta-analysis. Results of the meta-analysis using PACES data shows no significant effect in favor of either SIT or HIIT (Figure 6). In 6 out of seven studies, enjoyment level indicated by the PACES was comparable between HIIT and SIT. However, in the study of Astorino (Astorino et al., 2019) and the study of Foster (Foster et al., 2015) which used the EES, significantly higher enjoyment levels in HIIT compared to SIT (modified classic SIT and Tabata SIT) were reported.

Eight studies analyzed non-acute (i.e., intervention duration: 4-week to 16-week) enjoyment responses to SIT, while one out of eight studies only measured one time point after 4-week intervention. Most studies (*n* = 5) reported non-significant change in enjoyment level of SIT. Nevertheless, one study reported consistently decrease in enjoyment during an 8-week Tabata training (Foster et al., 2015); and one study reported that enjoyment level decrease significantly after the first week in RST while remained stable from the week 4 to week 12 (Hu et al., 2021).

## Discussion

Whilst there is a growing body of literature that recognized the substantial physiological benefits induced by SIT, there has been reported concerns about psychological responses of SIT protocols. This type of training involves supramaximal intensity and has been feared to be too physically demanding for individuals and especially inactive ones who often have low tolerance to high intensity exercise and have been presumed to have low intrinsic motivation to participate in any physical activity. These unsubstantiated claims must be scientifically and rigorously evaluated before making position statements. The current study aimed to synthesis the literature on the effects of SIT on psychological responses including two of the most reported variables—enjoyment and affect with respect to MICT and HIIT with near maximal intensity. Overall, the results from the included studies demonstrated a wide-range of affective valences and enjoyment scores to SIT. Affective responses decreased largely during SIT, with the lowest negative affective valences recorded during the majority of SIT studies. Affective valences were more negative immediately after exercise in SIT compared to MICT. It is important to note, however, that no significant differences were found between SIT and MICT in the meta-analyses in the lowest score and post-exercise score after recovery, indicating that the maximal adverse impact on affective responses of “all-out” intensity in SIT may not be as strong as generally assumed (Ekkekakis, 2003; Hardcastle et al., 2014). Levels of enjoyment after SIT were varied and inconsistent across studies that incorporated different protocol design and assessment times. The meta-analyses for comparisons in PACES scores indicated comparable enjoyment responses between SIT and MICT or HIIT.

Ratings of pleasure and displeasure through the FS could be largely impacted by exercise intensity according to previous studies in continuous training (Ekkekakis, 2008; Decker and Ekkekakis, 2017), in which the lowest point of affect valences could represent the magnitude of the impact. Negative affective valences observed in most of the studies (i.e., 9 out of 12 studies) during SIT suggests that SIT might lead to displeasure and discourage its participants. Interestingly, the lowest scores of affect valences were similar between SIT and MICT and lower in HIIT compared to SIT. Discrepancies in interval protocol design might influence perceived physical demand (i.e., RPE) (Borg, 1982) and lead to differences in affective responses. Significant associations between changes in RPE and changes in affect were found in a retrospective study that revisited the data collected in five previous studies from the same research team (Astorino and Vella, 2018). Consistent with the present review, positive affect valences were observed in SIT protocols (i.e., REHIT and RST) that induced low RPE (∼6 out of 10 in Brog CR-10 scale) (Niven et al., 2018; Astorino et al., 2020), while negative affect valences reported were usually accompanied by higher RPE (∼ 8–9 out of 10 in Brog CR-10 scale (Follador et al., 2018; Stork et al., 2018) or 17–18 out of 20 in Brog 6–20 scale (Marin et al., 2019; Marques et al., 2020). Moreover, adverse events (i.e., vomit) were reported in one study applied classic SIT with relatively high RPE (i.e., 16 ± 2.5), which led to seven or ∼14% participates drop-outs of the exercise protocol (Tritter et al., 2013). In this sense, lowering perceived exertions during SIT by adjusting the protocol design might help to mitigate negative affective responses and avoid adverse events. Specifically, low-volume SIT protocols with shortened sprint duration and fewer sprint repetitions as adopted in studies analyzing REHIT (i.e., 2 s × 20 s sprinting against 5% of BM + 3-min recovery) (Astorino et al., 2020) or RST (i.e., 10 s × 6 s sprinting against 7.5% BM + 60 s passive recovery) (Niven et al., 2018) could be less physically demanding and induce more positive affective responses, thus potentially increasing future exercise adherence.

Based on the scores of the PACES reported in the included studies, there was a neutral or above intermediate level of enjoyment responses to SIT regardless the protocol designs. It seems to be conflicting reports on enjoyment responses to SIT, considering the peak negative affective valences occurred frequently in most studies. Nevertheless, considerations of differentiating enjoyment as a subsequent affective judgment from general feelings during exercise as indicated by affect valences might help to settle the disagreements (Niven et al., 2020). Specifically, enjoyment measured by multi-item PACES post exercise indicate the recalled memory of enjoyment during previous exercise experiences, which is off-stimulus affective responses (Haile et al., 2016). In contrast, affect valences measured by single-item FS provide information of overall affective responses rather than specific affective domains. Moreover, items in the PACES tend to access more specific feelings such as energizing, stimulating, refreshing and sense of accomplishment (Kendzierski and DeCarlo, 1991) than general pleasure assessed by the FS. It is possible that some of these feelings (i.e., stimulating, feeling of accomplishment) might likely occur in SIT as perceived activations indicated by the FAS were reported high during SIT, and completion of more challenging SIT with “all-effort” could induce greater feelings of accomplishment compared to low- or moderate-intensity exercises.

Although comparable enjoyment responses between SIT and MICT were reported in several studies, cautions should be noted when generalizing the data from the existed studies that incorporated different study designs. In the study of Kriel et al. (2019), a lower enjoyment level analyzed through PACES in SIT with respect to MICT were reported. However, in order to match for the total mechanical work between the MICT group and the SIT group, the training durations of MICT (i.e., 5:33–7:38 min) in this study are considerably shorter than that of the other studies (i.e., 30–45 min) including MICT protocols. Thus, a lower training volume and significantly shorter work duration in SIT might explain the comparable enjoyment level compared to MICT with higher training volume and longer work duration. The study by Foster et al. (2015) also reported that SIT was significantly less enjoyable than MICT, which was indicated by lower EES scores recorded in the SIT group. However, the SIT protocol is defined as Tabata with recovery duration twofold shorter than interval duration. As such, this demanding work-to-rest ratio may aggravate the strenuousness for inactive individuals to perform SIT and induce a lower level of enjoyment.

Despite that the results gathered in the current review providing a summary of overall affective and enjoyment responses, individual differences still existed with a wide-range of affect valences and enjoyment scores to SIT. Identifying the exact variables that impact affective and enjoyment responses to dissect the mechanism behind the results could be challenging, yet several included studies tried to unravel the mystery of inconsistent psychological responses among individuals. One of the examined cognitive variables was tolerance of the exercise intensity. Two studies (Bradley et al., 2019; Marin et al., 2019) suggested that affective valences were influenced by self-reported tolerance measured through Preference and Tolerance for Exercise Intensity Questionnaire (PRETIE-Q) as affective valences were significantly lower (Bradley et al., 2019) or post-exercise fatigue (Marin et al., 2019) was higher in groups with lower levels of tolerance. However, one study pointed out that tolerance might not influence affective responses to REHIT (Astorino et al., 2020) which posed a relatively low physical demand (i.e., low RPE). The other cognitive parameter that has been analyzed was exercise self-efficacy which indicates the confidence to complete a given exercise protocol under specific conditions. Increasing self-efficacy through positive feedback provided by the experimenters stimulated higher levels of enjoyment according to the study of Tritter et al. (2013). Nevertheless, given the limited number of studies that analyzed cognitive parameters, more evidence is needed to confirm the associations between affective responses to SIT and cognitive parameters. The exercise environment (e.g., music background and exercise in groups) might also influence exercise experiences and psychological responses. Listening to music might mitigate the adverse feelings induced by SIT with supramaximal intensity as affective responses to SIT were more positive in music condition according to the studies of Stork et al. (2015, 2019). Moreover, exercising in groups might induce higher motivations and favorable affective responses to intense form of SIT (i.e., Tabata) as one study reported higher enjoyment in Tabata than MICT when performed in groups with music playing during workouts (Wilke et al., 2019).

In the present study, a sample size of a total of 763 healthy adult individuals from 25 studies were identified after completed search throughout the available databases. There were several research gaps and issues that worth of mention. Firstly, most of the studies (*n* = 23) in the current review only included young participants (i.e., < 30-year-old). The limited inclusion of young participants in SIT protocols suggests that future studies could benefit from involving various age groups to explore psychological responses in SIT and it was inappropriate to conclude that older subjects would respond to SIT similarly as the relatively young subjects from the current analysis. Secondly, most of the included studies analyzed acute responses to SIT (*n* = 14) or intervention duration of no more than 5 weeks (*n* = 3). Moreover, most interventions in the included studies were carried out in laboratory under supervision, which might ensure high adherence rate (i.e., 90–100%), while limited data from only two studies showed lower adherence (i.e., ∼70%) to SIT in unsupervised conditions. Therefore, data regarding long-term effects of SIT performing in “real-world” settings are still lacking, which highlight the need for more long-term studies to attain a comprehensive understanding of psychological responses and adherence to this training modality. Thirdly, there might be an issue generalizing affective and enjoyment responses across studies due to applying varied methods of differing intensity in SIT and overlooking individual differences. For example, only a small number of studies determined exercise intensity based on ventilatory threshold (VT) or other metabolic landmarks which has been suggested as a more appropriate method with individual differences taken into consideration (Marin et al., 2019). Nevertheless, the measurements of these metabolic landmarks could be impractical in a real-life setting. Fourth, cognitive and environmental variables, which might vary to a large extent between people, were not often controlled in the included studies, indicating a need for more studies with individual differences taken into consideration and appropriated measurements in this area of research. Lastly, there was a limited number of studies (5 out of 28 studies) that analyzed behavior-related parameters such as intention and preference. Despite that the results indicated certain SIT protocol (i.e., RST) was preferred and could induce higher intention compared to classic SIT and HIIT, more studies are needed to investigate the influences of differences in the design of SIT protocols on behavior-related parameters and actual exercise behavior.

The current review is the first study that tried to synthesize the existing studies on affective and enjoyment responses of SIT with respect to differences in exercise configuration in healthy individuals. However, there are a few limitations in the present study that should be mentioned. Firstly, categorizations of SIT protocols based on differences in protocol design might not be precise, since small variations in work-to-rest ratio across studies still existed. Moreover, apart from differences in protocol design, there were other factors that could influence affective and enjoyment responses awaiting to be investigate. Lastly, several outcomes in the meta-analysis resulted in a high degree of heterogeneity and unbalanced weight across studies with greater impact from some studies (Niven et al., 2018; Stork et al., 2018), which might impact the strength of the results.

Overall, the results in this review demonstrated that SIT elicits negative and lower affective valences compared to MICT immediately post-exercise, but similar low affective valences during exercise and comparable post-exercise affective state as MICT or HIIT. Moreover, enjoyment responses in SIT were comparable to MICT or HIIT in healthy individuals, suggesting similar future adherence in SIT with better time-efficiency than MICT or HIIT. Based on the available literature and data at this time, it is still premature to conclude on whether SIT, or which SIT configuration, could induce more positive affective responses. Likewise, there is inadequate data to date to be able to conclude which exercise modality is going to be more likely to be adhered due to the limited age groups included and inconsistent research design across studies involving SIT protocols, and individual differences existed among the participants. Our findings suggested that it might be important to consider adopting low-volume SIT protocols with shorter sprint duration and fewer sprint repetitions (e.g., REHIT or RST) and incorporating music, group exercise to reduce adverse affective responses, especially when introducing SIT to individuals with low tolerance to high intensity exercise. Moreover, given that physiological improvements elicited by SIT is not attenuated with shorter and fewer sprints according to previous meta-analysis (Vollaard et al., 2017), low-volume SIT that produced more positive affective responses and possibly higher future adherence could be a better choice to maximize the time-efficiency of SIT.

## Data Availability Statement

The original contributions presented in the study are included in the article/Supplementary Material, further inquiries can be directed to the corresponding author/s.

## Author Contributions

MH and ZK contributed to conception and design of the manuscript, performed data search, and data extraction. MH performed data-analysis. MH, MJ, JN, and ZK drafted and revised the manuscript. All authors contributed to the article and approved the submitted version.

## Conflict of Interest

The authors declare that the research was conducted in the absence of any commercial or financial relationships that could be construed as a potential conflict of interest.

## Publisher’s Note

All claims expressed in this article are solely those of the authors and do not necessarily represent those of their affiliated organizations, or those of the publisher, the editors and the reviewers. Any product that may be evaluated in this article, or claim that may be made by its manufacturer, is not guaranteed or endorsed by the publisher.
