# Supplementary material for: Affective and Enjoyment Responses to Sprint Interval Training in Healthy Individuals: A Systematic Review and Meta-Analysis
Source: Front Psychol. 2022 Mar 9;13:820228. doi: 10.3389/fpsyg.2022.820228 (PMC8959769; doi:10.3389/fpsyg.2022.820228)
Supplement: Supplementary file 1 [file Table_1.docx]

**Supplementary Table 1** Study design and findings of psychological responses in the included studies

| **Study** | **Design** | **SIT protocol** | **Compared protocol** | **Intervention Duration** | **Setting** | **Findings on psychological responses of the research** |
| --- | --- | --- | --- | --- | --- | --- |
| Astorino et al.  (2019) | Randomized between subjects; | ***Cycling****:*  *PER (Periodized interval training):*  week 1, 4:  *HIIT* - 10 × 60 s at 80-85% of PPO + 60 sec recovery  ***Duration****:*  10 min work + 10 min rest  week 2, 5:  *Classic* *SIT* - 6 × 20 s sprinting at 105% PPO + 120 s recovery  ***Duration****:*  2 min work +12 min rest  week 3, 6:  *HIIT* (high volume) - 7 × 120 s at 60% PPO + 60 sec recovery  ***Duration****:*  14 min work + 7 min rest | *HIIT*:  10 × 60 s at 80-85% of PPO + 60 s  recovery  ***Duration****:*  10 min work + 10 min rest | 3×/week for 6 weeks | Two sessions  in laboratory and 1 session outside of the laboratory per week | · **Enjoyment** (PACES) (measured 5 min after completion of the first session each week) was significantly lower in PER vs. TRAD every week. In PER, higher PACES values were found in SIT (week 2, 5) vs. HIIT and HIIT (high volume) (week 1,3 and week 4,6). The lowest PACES values were observed in PER during HIIT (high volume) in weeks 3 (73.1 ± 16.9) and 6 (76.4 ± 16.6).  · **Affect** (FS) (measured immediately end-exercise) was lower during PER in weeks 1, 3, 4, and 6 of training (HIIT and HIIT (high volume)) vs. TRAD (HIIT). Declines were found in both groups with significantly lower values end-exercise vs. warm-up, yet lower mid-point affect vs. warm-up was also found in PER during HIIT and HIIT (high volume). Lowest and negative affect was observed in PER during HIIT (week 1) end-exercise (−0.2 ± 1.2). |
| Astorino  et al.  (2020) | Between subjects; | ***Cycling****:*  *REHIT:*  *Group: Subjects below average VO_2max_*  2 × 20 s “all-out” sprint at 5% body mass + 180 sec active recovery  ***Duration****:*  40 s work + 6 min rest | *Group:*  *Subjects above average* *VO_2max_:*  same REHIT protocol | Acute | Laboratory | · **Enjoyment** (PACES) (measured 5 min post training) was not different between groups (93.2 ± 20.8 vs. 91.1 ± 16.4).  · **Affect** (FS scores) were consistently higher (measured pre-, during, and 10-min post training ) in subjects above average VO_2max._ Declines were found in both groups after warm-up and was lowest immediately after sprint 2 with scores (above average VO_2max_ (1.4 ± 1.9) and below (1.1 ± 2.0).  · **Tolerance** **and** **Preference** was not different between groups. Tolerance was not correlated to perception of exercise. Preference was significantly higher in subjects above average VO_2max_ |
| Bradley  et al.  (2019) | Between subjects; | *Cycling:*  *RST:*  *Group: HT-high tolerance subjects*  10 × 6 s sprinting against 7.5% BM (males) or 6.5% BM (females)+ 60 s active recovery (50-59 sec unload, 59-60 sec unload sprinting)  ***Duration****:*  1 min work + 10 min rest | *Group:*  *LT-low tolerance:*  same SIT protocol  *VLT-very low tolerance:*  same SIT protocol | Acute | Laboratory | · **Affect** (FS) was significantly lower in VLT vs. LT and HT at all the time points (measured after sprint 2, 4, 6, 8, 10, and 20-min post training). Negative affect responses were found in VLT (~ -0.5) in bout 8 and 10 but not in LT and HT. No significant differences between LT and HT (~ 2-3).  · **Arousal** (FAS) did not differ significantly among the groups (~ 3 vs. ~ 4). Significantly increasing between baseline and sprint 2 and decreasing from sprint 10 to 20 min post exercise were found.  · **Circumplex** (FS+ FAS): tiredness found in sprints 8 and 10 in VLT. LT and HT generated state of energy from sprints 4-10. VLT and LT returned to calmness post training, while HT remained state of energy.  **· Self-efficacy:** exercise task self-efficacy was not significantly influenced by tolerance. |
| Follador  et al.  (2018) | Randomized within subjects; | *Cycling:*  *Classic SIT :*4 × 30 s sprinting against a resistance of 7.5% of BM+ 270 s active recovery at a low cadence (<50 rpm) against a resistance of 30 W  ***Duration****:*  2 min work + 18 min rest  *Tabata:* 7 × 20 s at a pedal cadence of 90 rpm + 10 sec passive recovery  ***Duration****:*  2 min 20 s work + 1 min 10 s rest | *HIIT:* 10 × 60 s at a pedal cadence of 80 rpm + 60 sec recovery  ***Duration****:*  10 min work + 10 min rest | Acute | Laboratory | · **Affect** (FS) (measured 10 min post training- “session affect”) values were the lowest in SIT (Tabata) (−1.1 ± 2.5) vs. SIT (Wingate) (0.4 ± 2.9) and HIIT (2.1 ± 2.0) during cycling, and were the lowest in SIT (vVO2max) (−0.4 ± 2.2) vs. HIIT (RPE) (0.9 ± 2.3) and HIIT (2.1 ± 1.8) during running. |
|  |  | *Running:*  *SIT (vVO2max):*  5 × 50% tlimvVO_2max_ at 100% vVO_2max_ + active recovery at 50% tlimvVO2max at 60% vVO_2max_ | *HIIT:* 4 × 4 min running at 90% to 95% HRmax + 3-minute active recovery runs at 70%HRmax  ***Duration****:*  16 min work + 12 min rest  *HIIT (RPE):* 4 × 1,000 meter at an intensity corresponding to a RPE of 8 (‘‘Hard’’) + active recovery at a RPE of 2 (‘‘Easy’’) |  |  |  |
| Foster et al. (2015) | Randomized between subjects; | *Cycling:*  *Tabata*: 8 × 20 s at 170% aerobic power +10 s active recovery (unload)  ***Duration****:*  2 min 40 s work + 1 min 20 s rest | *MICT*: 20 min at V̇O_2_ equal to 90% VT;  ***Duration****:* 20 min  *HIIT:* 13 × 30 sec at 100% aerobic power + 60 sec active recovery at 90% VT  ***Duration****:*  6 min 30 s work + 13 min rest | 3×/week for 8 weeks | Laboratory | · **Enjoyment** (EES) values (measured pre-, during and post training on baseline, weeks 2,4,6,8) significantly decreased across weeks in all groups and were significantly lower in SIT (Tabata) group (~2.5 out of 7) vs. MICT and HIIT. The EES was lower during training than either before or after training. |
| Hu et al. (2021) | Randomized between subjects; | *Cycling:*  *RST:*  80 × 6 s sprinting against initial workload 1.0 kg (increased by 0.5 kg increment if completed two consecutive sessions) interspersed with 9 s rest  ***Duration****:*  8 min work + 12 min rest | *MICT:*  ~ 65 min at 60% VO_2peak_  ***Duration****:* ～65 min  *HIIT:*  4 min at 90% VO_2peak_ followed with 3 min recovery for ~ 60 min  (mechanical work matched with MICT)  ***Duration****:* ～60 min | 3×/week for 12 weeks | Laboratory | **· Enjoyment** (PACES) (measured immediately after the completion of the first and the last training sessions of the 4th, 8th and 12th week) did not vary between protocols (86~100). The PACES score decreased from week 1 to week 4 in both SIT (p < 0.05) and HIIT groups. In contrast, the enjoyment response to the MICT intervention remained relatively stable throughout the study. |
| Kriel,  et al.  (2018) | Randomized within subjects; | *Cycling*  *Classic SIT:* 4 × 30 s sprinting + 120 sec passive recovery  ***Duration****:*  2 min work + 8 min rest |  | Acute | Laboratory | **· Enjoyment** (PACES) (measured within 5 min post training) values were significantly higher in SIT (running) vs. SIT (cycling) (74.5 vs. 91.46) |
|  |  | *Running:*  *Classic SIT*: 4 × 30 s at maximal speed (with an initial running speed of 8 km·h-1increasing by 1 km·h-1 every 30 s until volitional cessation) + 120 s passive recovery  ***Duration****:*  2 min work + 8 min rest |  |  |  |  |
| Kriel,  et al.  (2019) | Randomized within subjects; | *Cycling:*  *Classic SIT*: 4 × 30 s sprinting + 120 sec passive recovery  ***Duration****:*  2 min work + 8 min rest | *MICT*: 5:33–7:3 min at 50% of the peak power output  (Mechanical work was matched with SIT)  ***Duration****:*  5:33-7:38 min | Acute | Laboratory | **· Enjoyment** (PACES) (measured within 5 min post training) values were significantly higher in MICT vs. SIT (~83 vs. ~93) |
| Marin  et al.  (2019) | Randomized within subjects; | *Battling rope exercise:*  *Classic SIT:* 4 × 30 s sprinting + 90 s recovery  ***Duration****:*  2 min work + 6 min rest  *SIT (15 s):* 8 × 15 s sprinting + 45 sec recovery  ***Duration****:*  2 min work + 6 min rest  *RST: 12* × 10 s sprinting + 30 sec recovery  (SIT protocols were work-matched)  ***Duration****:*  2 min work + 6 min rest |  | Acute | Laboratory | · **Enjoyment** (PACES) (measured 15 min after each session) values was not different between protocols (77-78 out of 119 total, one item removed)  · **Affect** (FS) measured pre-, during (-25%, 50%, 75%, 100% quartile) and 15-min post training did not vary between protocols. Lowest affect valences were -0.36 ± 3.41, -0.43 ± 3.75, -0.93 ± 3.49 in response to RST, SIT (15 s) and Classic SIT. Results revealed a significant decline in all three protocols.  **· Arousal** (FAS) (measured at the same time as Affect valence) did not differ significantly between protocols. There was a progressive increase in FAS.  **· Self-efficacy** did not vary between protocols. The indirect effect of changes of FS intermediated by self-efficacy was statistically significant. There was positive correlation between enjoyment and the self-efficacy. |
| Marques  et al.  (2020) | Randomized within subjects; | *Running:*  *RST*: 19 × 6 s sprinting + 40 s active recovery (30% of maximal aerobic speed)  ***Duration****:*  1 min 54 s work + 12 min 40 s rest  *Classic SIT*: 4 × 30 s sprinting + 240 sec active recovery (30% of maximal aerobic speed)  ***Duration****:*  2 min work + 16 min rest | *HIIT (long interval)*: 10 × 60 sec at 100% of maximal aerobic speed + 40 s active recovery (50% of maximal aerobic speed)  ***Duration****:*  10 min work + 6 min 40 s rest  *HIIT (short interval):* 10 × 30 s at 120% of maximal aerobic speed + 120 s active recovery (30% of maximal aerobic speed)  ***Duration****:*  5 min work + 20 min rest | Acute | University’s outdoor sports facilities  (supervised) | **·** **Enjoyment** (PACES) (measured post training) values did not vary between protocol or sex(87 vs. 89).  · **Affect** (FS) measured pre-, during (-25%, 50%, 75%, 100% quartile) and 20-min post training (20 quartile) did not vary between protocol or sex. Affect decreased across protocols (-0.5 vs. -0.7 Classic SIT vs. RST). Specifically, higher values pre-exercise and at the first quartile compared with all other time points were found. Higher values at midpoint compared with the last two quartiles and higher values 20 minutes after exercise compared with the last two quartiles were found.  · **Preference** was higher in SIT (RST) vs. the other three protocols. |
| McKie  et al.  (2018) | Randomized between subjects; | *Running:*  *Classic SIT:* 4-6 × 30 s sprinting + 240 sec recovery  ***Duration****:*  2 min work + 16 min rest,  3 min work + 24 min rest  *SIT (15 sec)*: 8-12 × 15 s sprinting + 120 sec recovery  ***Duration****:*  2 min work + 16 min rest,  3 min work + 24 min rest  *RST:* 24-36 × 5 s sprinting + 40 sec recovery  ***Duration****:*  2 min work + 16 min rest  3 min work + 24 min rest | *CON:* no-exercising control | 3×/week for 4 weeks | Laboratory | **· Enjoyment** (PACES) (measured immediately after the last training session) values did not vary between protocols (65.2 ± 6.1 (5s), 62.2 ± 17.6 (15s), 64.2 ± 5.1 (30s). (119 total, one item removed).  *Two items added:*  1. how much did you enjoyed? 6.7 ± 2.1(5s), 7.9 ± 1.3(15s), 6.4 ± 2.4(30s)  2. Enjoyment performing: 6.5 ± 1.8(5s), 6.7 ± 1.4(15s), 5.6 ± 2.2(30s)  **· Self-efficacy** did not vary between protocols, but the ability to avoid over-exertion were significantly higher in SIT (5s) compared to SIT (30s). |
| Metcalfe  et al.  (2020) | Randomized control group | *Cycling:*  *REHIIT:* 2 × 10 s (session 1-3) or 15 s (session 4-6) or 20 s (session 7-12) sprinting against resistance equivalent to 5% of BM + 180s active recovery (~25w)  ***Duration****:*  20 s work + 6 min rest or  30 s work + 6 min rest or  40 s work + 6 min rest | *CON:* no-exercising control | 2×/week for 6 weeks | Workplace | **· Enjoyment** (PACES) (measured after the 6-week intervention) was 89.1 ± 16.6. Enjoyment and expected future enjoyment were high.  **· Self-efficacy** in the training group was 7.8 ± 1.2 (Highest score: 9). |
| Niven  et al.  (2018) | Randomized within subjects; | *Cycling:*  *RST*: 10 × 6 s sprinting against 7.5% of BM + 60 s passive recovery  ***Duration****:*  1 min work + 10 min rest | *MICT*: 30 min at PO equal to 85% of VT  ***Duration****:* 30 min  *MVCT*: ~23 min at PO equal to 105% of VT (work done in matched that in MICT)  ***Duration****:* ~23 min | Acute | Laboratory | **· Affect** (FS) measured pre-, during (every 20% of exercise time) and 15 min post training remained positive and did not vary among protocols. The largest reduction (warm-up to 100% of exercise) during the training in affective valence occurred in MVCT (−1.75 ± 2.42) followed by SIT (−1.17 ± 1.99).  **· Arousal** (FAS) did not differ significantly between SIT and MVCT during the training, but lower in MICT (~2.8) vs. SIT (~4.5) throughout exercise. Higher values in MVCT vs. MICT at 60% 80% and 100% of the exercise.  **· Circumplex** showed a consistently state of calmness in MICT. In SIT, there were high activation and positive affect (energy) throughout the exercise, and immediately following exercise, calmness generated. In MVCT, calmness was found prior to exercise and for the first 40% of exercise; a sense of energy generated from 60%-100% of exercise and returned to calmness post training. |
| Olney  et al.  (2018) | Randomized within subjects; | *Cycling:*  *Classic SIT*: 6 × 20 s at intensity of 140% W_max_ +75 s active recovery at intensity of 20% W_max_  ***Duration****:*  2 min work + 7 min 30 s rest | *MICT*: 25 min at intensity of 40% W_max_  *HIIT (short interval):* 8 × 60 s at 85% W_max_ + 75 s active recovery at 20% Wmax  ***Duration****:* 25 min  *HIIT (long interval):* 6 × 120 s at 70% W_max_ + 60 s active recovery at 20% W_max_  ***Duration****:*  12 min work + 6 min rest | Acute | Laboratory | **· Enjoyment** (PACES) (measured 10 min post training) values did not vary among protocols (85.2 ± 17.3 (HIIT) vs. 87.6 ± 21.2 (SIT). Despite no gender differences between protocols were observed, women demonstrated higher PACES scores vs. men  **·** **Affect** (FS) measured during (at the end of the respective bout in SIT/HIIT and at 6.25, 12.5, 18.75, and 25 minutes in MICT) was significantly more positive in MICT (3.30 ± 0.94) at 50, 75, and 100% of session duration vs. HIIT or SIT (-0.95 ± 2.50)., with no significant differences between these two regimes. Affect gradually declined throughout SIT and HIIT regimes but maintained in MICT after 25% of the exercise. There were no significant different responses between genders. |
| Rowley  et al. (2017) | Randomized between subjects; | *Running:*  *Classic SIT*:  4 (week1-3), 6 (week4-6), 8 (week7-9), 10 (week10-12) × 30 s +240 s passive recovery  ***Duration****:*  2 min work + 16 min rest  3 min work + 24 min rest  4 min work + 32 min rest | *MICT*: 30 min (week1-3), 40 min (week4-6), 50 min (week7-9), 60 min (week10-12) at an intensity of their individual 45–55% HRR  ***Duration****:*  30 min, 40 min, 50 min, 60 min | 3×/week for 12 weeks | Laboratory | **· Enjoyment** (PACES) values (measured post training on baseline, weeks 6,12) did not vary between protocols. The SIT group experienced a non-significant increase in perceived enjoyment over 12 weeks (baseline: 58.6 ± 3.2; 6 weeks: 61.2 ± 5.9; 12 weeks: 64.0 ± 5.1), whereas MICT exhibited a non-linear trend (baseline: 58.8 ± 4.1; 6 weeks: 64.4 ± 2.4; 12 weeks: 60.6 ± 5.2). |
| Schaun and Alberton (2020) | Randomized between subjects; | *Whole body exercise:*  *Classic SIT:*  8 × 20 s sprinting (burpees, mountain climbers, squat & thrusts with 3.1 kg dumbbells, jumping jacks) +10 s passive recovery  ***Duration****:*  2 min 40 s work + 1 min 20 s rest |  | 3×/week for 16 weeks | School’s exercise facility (supervised) | **· Enjoyment** (PACES) values (measured 10 min post training on weeks 1, 5, 9, 13, 16) did not vary among protocols (~95). |
|  |  | *Running:*  *Classic SIT:*  8 × 20 s at 130% of the velocity associated with the second ventilatory threshold (VT_2_) + 10 s passive recovery  ***Duration****:*  2 min 40 s work + 1 min 20 s rest | *MICT:* 30 min at 90–95% heart rate associated with VT_2_  ***Duration****:* 30 min |  |  |  |
| Sim et al. (2014) | Randomized within subjects; | *Cycling:*  *SIT*: 15 s at 170% VO_2peak_ + 60 s active recovery at 32% VO_2peak_  ***Duration****:* Not reported (30-minute exercise session) | *MICT*: 30 min at 60% VO_2peak_  (mechanical work was matched with SIT protocols)  ***Duration****:* 30 min  *HIIT*: 60 sec at 100% VO_2peak_ + 240 sec active recovery at 50% VO_2peak_  ***Duration****:*  Not reported (30-minute exercise session) | Acute | Laboratory | **· Enjoyment** (PACES) values (measured 10 min after each training session) did not vary among protocols (82±17 (SIT), 86±11 (HIIT), 85±13 (MICT). |
| Songsorn et al. (2020) | Randomized within subjects; | *Cycling:*  *REHIT:* 2 x 20s ‘all-out’ cycle sprints at 7.5% body mass  ***Duration****:*  40 s work + 9 min 20 s rest (10-minute exercise session) | *MICT:* 30 min at 40% Wmax  ***Duration****:* 30 min  *HIIT:* 10 x 60s at 100% Wmax)  ***Duration****:*  10 min work + 12 min rest (22-minutes exercise session) | Acute | Laboratory | **Affect** (FS) (meassured before exercise, every 2 min during exercise, and then 10- and 30-min post-exercise. There were no group differences in change in affect (MICT: −2.1 ± 1.6; HIIT: −3.9 ± 1.9; REHIT: −2.7 ± 3.0), while higher lowest affect in MICT and SIT compared to HIIT. (MICT: 0.7 ± 1.4; HIIT: −1.3 ± 2.8; REHIT: 0.6 ± 2.4)  **Enjoyment** (PACES) (measured 30 min post- exercise) were not significant different across the exercise conditions (MICT: 70 ± 16 vs HIIT: 80 ± 19 vs REHIT: 87 ± 22). |
| Stork et al. (2015) | Randomized within subjects; | *Cycling:*  *Classic SIT with self-selected music:* 4 × 30 s sprinting 7.5% of whole-body weight + 4 min of rest.  ***Duration****:*  2 min work + 16 min rest | *CON: no music:* same SIT protocol | Acute | Laboratory | **· Enjoyment** (PACES) values (measured immediately after exercise and at 30 and 60 min postexercise) significantly increased over time and was consistently higher in the music condition (85-90 (Music), 79 - 83 (No music)).  **· Affect** (FS) (measured pre-, during- every sprint bout and rest, and post exercise) were not different between conditions (~0.2 (Music), ~ -0.3 (No Music). FS score decreased across all four bouts and across all four rest periods. |
| Stork et al. (2018) | Randomized within subjects; | *Cycling:*  *REHIT:* 3 × 20 s sprinting against 5% of whole-body weight +120 s passive recovery;  ***Duration****:*  1 min work + 6 min rest | *MICT*: 45 min at intensity of 35% W_max_  ***Duration****:* 45 min  *HIIT*: 10 × 60 s at 70% W_max_ + 60 s passive recovery  ***Duration****:*  10 min work + 10 min rest | Acute  +4-week follow-up (recording subsequent exercise behavior) | Laboratory | **· Enjoyment** (PACES) (measured immediately after the training session) values did not vary between protocols (83.70 ± 19.20 (MICT), 84.43 ± 18.47 (HIIT), 81.63 ± 18.78 (SIT).  **· Affect** (FS) measured pre-, during (after each bout in HIIT and MICT and every 5 min in MICT) and 10 min and 20 min post training. Affect valence declined during all three training regimes, while there was a larger decrease in SIT (3.92 ± 1.28). Peak negative FS was more negative for SIT than MICT despite no significant differences were reported (2.86 ± 0.90 vs. 3.84 ± 1.28). Affect responses immediately postexercise was more positive for HIIT than SIT despite non significantly differences, but no significantly among the three groups differences were found 10- or 20-min postexercise  **· Arousal** (FAS) was lower during MICT vs. SIT and HIIT, with no difference between these two protocols. Postexercise FAS scores were similar across the three groups.  **· Preference** was similar across the three groups. |
| Stork et al. (2019) | Randomized within subjects; | *Cycling:*  *REHIT with motivational music*  3 × 20-s sprinting against 5% of whole-body weight + 120 s recovery periods  ***Duration****:*  1 min work + 6 min rest | *Group:*  *Podcast control:*  same SIT protocol  *No-audio control:*  same SIT protocol | Acute | Laboratory | **· Enjoyment (PACES)** (measured immediately after the training session) values were significantly higher in the motivational music condition (89.58 ± 17.33 (Music), 83.92 ± 19.49 (Podcast), 85.28 ± 17.92 (No-audio).  **· Affect** (FS) measured pre-, during (at end of/immediately following sprints 1, 2, and 3, and during the last ∼35 s of recovery periods) and immediately, 5-min and 10-min post training were not significantly different across the conditions. Lowest valences ranged from ~ -0.4 (No-audio) to ~ 0.2 (Music or Podcast). Decrease of affect valences from pre-training to sprint 3 were observed in all the conditions, while increased significantly from pre-exercise to all other time points during exercise across all conditions.  **· Arousal** (FAS) measured with FS were not significantly different across the conditions.  **· Liking** (one-item 10-point scale) scores were higher during the music condition. |
| Townsend et al. (2016) | Randomized within subjects; | *Running:*  *Classic SIT:* 4 × 30 s sprinting + 240 sec recovery  ***Duration****:*  2 min work + 16 min rest  *SIT:* 8 × 15 s sprinting + 120 s recovery  ***Duration****:*  2 min work + 16 min rest  *RST:* 24 × 5 s sprinting + 40 sec recovery  ***Duration****:*  2 min work + 16 min rest |  | Acute | Laboratory | **· Enjoyment** (PACES) (measured 30-min after the training session) was significantly higher in SIT (5 sec) and SIT (15 sec) (no differences between these two) vs. SIT (30 sec) (96.6 ± 1.0 (5s), 88.6 ± 1.4 (15s), 83.4 ± 1.6 (30s) (119 total, one item removed).  *Two items added:*  1. How much did you enjoyed? 7.11 ± 1.17 (5s), 6.44 ± 2.4 (15s), 4.56 ± 2.54(30s)  2. enjoyment performing: 5.67± 2(5s), 4.89 +2.26(15s), 3.11 +2.21(30s)  **· Affect** (FS) measured pre-, during (at 4.5, 9, and 13.5 min), immediately and 30 min post training. Affect valence were significantly higher in SIT (5 sec) vs. SIT (30 sec) after mid-point of the training and 30 min postexercise (-0.9 ± 1.9 (5s), -1.9 ± 2.5 (15s), -2.8 ± 2.5 (30s).  **· Self-efficacy** was significantly higher in SIT (5 sec) and SIT (15 sec) (no differences between these two) vs. SIT (30 sec).  **· Intentions** was significantly higher in SIT (5 sec) vs. SIT (15 sec) and SIT (30 sec).  **· Preference** all participants verbally selected the 5:40 exercise protocol over the other protocols. |
| Tritter  et al. (2013) | Randomized between subjects; | *Running*  *Classic SIT (HE-subjects with high efficacy feedback)*  4 × 30 s sprinting + 240 s passive recovery  ***Duration****:*  2 min work + 16 min rest | *Group:*  *LE- low efficacy feedback:*  same SIT protocol  *Control - no efficacy feedback):*  same SIT protocol | Acute | Laboratory | **· Enjoyment** (PACES) (measured 5-min after the training session) was significantly higher in SIT (HE) vs. SIT (LE) and control groups (86.188± 17.966, range: 52.94-120.71 (HE), 66.107 ± 23.5, range: 27.53-109.06 (LE), 72.433, ± 18.024, range: 27.53-101.65 (No feedback).  **· Affect** **(**SEES) variables were similar among the groups, yet greater decrease in positive well-being and increase in distress were found in SIT (LE).  **· Self-efficacy** declined in all groups, but SIT6 (HE) had the smallest decrease in self-efficacy vs. the control and SIT (LE) groups. |
| Wilke  et al. (2018) | Randomized between subjects; | *Whole body exercise*  *Tabata SIT:* 30×20 s all‐out bouts with 10s breaks (plus 1-min rest every 10-set in week 1 or every 15-set in week 2)  ***Duration****:*  10 min work + 5 min rest | *MICT:* walking for 50 minutes at 50% HRR (weeks 1 and 2), to 55% HRR (weeks 3 and 4) and 60% HRR (weeks 5 and 6)  ***Duration****:* 50 min | 3×/week for 6 weeks | Outdoors (supervised) | **· Enjoyment** (five‐level Likert scale) was significantly higher in SIT (~4) than MICT. |
| Wood  et al. (2016) | Randomized within subjects; | *Cycling:*  *Classic SIT*: 8 × 30 s sprinting at intensity of 130% W_max_ + 90 s active recovery at 25% W_max_  ***Duration****:*  4 min work + 12 min rest | *HIIT*: 8× 60 s at 85% W_max_ + 60 sec active recovery at 25% Wmax  ***Duration****:*  8 min work + 8 min rest | Acute | Laboratory | · **Affect** (FS) (measured immediately after sprint 2, 4, 6, 8) did not vary between protocols. Affect valence declined during both protocols. There was a tendency for more positive affect at cessation of HIIT compared with SIT (-1.0 + 2.4 (HIIT), -2± 2.5 (SIT). |

~: estimated scores extracted from figures

EES = Exercise Enjoyment Scale; F = female; FAS = Felt Arousal Scale; FFM = fat-free mass; FS = Feeling Scale; HIIT = high-intensity interval training; HR = heart rate; HRR = reserve HR_max_ = maximum heart rate; MICT = moderate-intensity continuous training; PACES = Physical Activity Enjoyment Scale; PANAS = Positive and Negative Affect Schedule, PO = power output; PPO = peak power output; REHIT: reduced exertion high intensity interval training; SEES = The Subjective Exercise Experiences Scale; SIT = sprint interval training; tlimvVO_2max_ = time limiter for the velocity at VO_2max_; VO_2peak_ = peak oxygen consumption; VT = ventilatory threshold; Wmax = maximum wat
